# Notes and News

**Published:** 1888-12-29

**Authors:** 


### NOTES AND NEWS.

COLLECTED AND COMMUNICATED.

We are obliged to hold over Chapter XIII. of our Serial Story, "False Impressions," until next week, owing to the exceptional pressure on our space.

On December 18th a new Convalescent Home for Children was opened at Gilmerton, N.B., by the Rev. Dean Montgomery. The home is very plain but comfortable, and was erected from plans prepared by Mr. Duncan, of Blairquhosh. There are three dormitories, containing 22 cribs in all; there is a nice large play-room. The building has cost £1,330, of which £200 yet remains to be defrayed.

The senior medical students at Edinburgh are ashamed of the late disturbances in the class-rooms, connected with the boycotting of Sir Morell Mackenzie. They have held meetings and expressed their strong disapproval of these manifestations, and they have also condemned the exaggerated accounts which have appeared in the public press. It seems strange to hear of Scotch medical students holding solemn conclaves in favour of law and order.

A special meeting of the Council of the Charity Organization Society was held last week, to hear a paper by Mr. Van de Linde on "The Preparation and Audit of Charity Accounts." The evil habit of amateur auditing of charity accounts is luckily on the wane, and the public is awaking to the knowledge that they ought to know how money given in charity is spent. Mr. Van de Linde's paper was eminently practical, and full of useful hints as to the best methods of dealing with donations, dividends, and other sources of income. The special representative committee have undertaken to consider the paper, and to advise what action the society should take with regard to carrying out its suggestions.

**The following vacancies are announced this week, the amount of salary in each case being given after the name of the institution:—**

**Resident Medical Officers.**

City of London Hospital for Diseases of the Chest, Victoria Park, N.E. Resident Clinical Assistant.

Consumption Hospital, Brompton. House Physician.

Derbyshire General Infirmary. Resident Assistant House Surgeon. —Board: no salary but bonus of £10 for six months.

General Hospital, Birmingham. Assistant House Surgeon — Board, no salary.

General Hospital for Sick Children, Pendlebury, Manchester. Junior Resident Medical Officer.—£80 per annum, with board, etc.

Hospital for Sick Children, Great Ormond Street, W.C. House Surgeon.—£50 per annum, with board, etc.

Liverpool Dispensaries. Assistant Surgeon.—£80 per annum, with board, etc.

Nottingham General Dispensary. Senior Resident Surgeon.— £180 per annum.

Royal Hospital for Children and Women, Waterloo Bridge Road, R E. Resident Medical Officer.—£70 per annum, with board, etc.

Darenth Schools for Imbecile Children. Assistant Medical Officer.—£120, board, etc.

**Non-resident Medical Officers.**

Ballymena Dispensary.—£120 per annum, and fees.

Evelina Hospital for Sick Children, Southwark Bridge Road, S.E.—Physician to Out-patients.

Evelina Hospital for Sick Children Registrar and Chloroformist. —£30 per annum.

Holloway and North Islington Dispensary. Honorary Surgeon to Rupert Road branch.

Leicester United Friendly Societies Medical Association, High-cross Street, Leicester. Medical Officer.—£100 per annum, with unfurnished house.

Salford Royal Hospital. Honorary Medical Officer for Pendleton Branch Dispensary.

The Coventry Hospital shows its worth by the excellent patronage it receives. Mrs. Gulson always speaks good of the hospital from personal experience, and points out the significance of the medical statistics. The committee have very properly named the new girls and children's wards, which owe their existence to Mrs. Gulson's exertions and munificence, the Gulson Wards.

The annual meeting of the subscribers to the Dublin Orthopædic Hospital took place on December 17th, the Right Hon. Mr. Justice Monroe presiding. Mr. Fisher read the report, in which it was stated that 105 in-patients had been benefited during the year. The receipts amounted to £905, and the hospital has just managed to keep out of debt, but only by keeping unoccupied 14 out of the 35 available beds.

The *Times* has started a correspondence on "Our London Hospitals and their Annual Deficit." A certain Mr. Coote came forward first, with the simple but Utopian scheme of penny-a-week subscriptions from the whole population. Of course, hospital secretaries and employers of workmen are all down on Mr. Coote, and desire to point out how unpractical is his plan. The strange thing is, how many schemes are always being propounded for paying off the National Debt and meeting other financial difficulties, but how seldom the wildest enthusiasts ever carry their theories farther than a letter to the *Times*.

At a meeting of the delegates of the Hospital Saturday Fund, held at the board-room, 41, Fleet Street, on Saturday, 22nd inst., under the presidency of Mr. W. G. Bunn, it was unanimously resolved to distribute the sum of £10,000 among 78 hospitals, 38 dispensaries, and 23 convalescent homes and surgical aid societies. The largest awards to hospitals were: London, £578 6s.; Brompton, £542 11s.; Guy's, £395 19s.; City of London for Diseases of the Chest, £263; St. George's, £259 5s.; University College, £246 15s.; St. Mary's, £242 1s.; Westminster, £183 2s.; Middlesex, £201 6s.; Royal London Ophthalmic, £181; Royal Free, £174 19s. 4d.; Charing Cross, £151 10s.; King's College, £144 3s.; North London for Consumption, £132 14s.; Hospital for Sick Children, £132 8s.; Seamen's, £128 10s.; German, £110 14s.; Royal, for diseases of the chest, £107 4s.; Tottenham Hospital, £96 17s.; St. John's, for diseases of the skin, £90 3s.; Victoria, for children, £88 2s.; Cancer, £100 13s.; North-West London, £92; Royal National for Consumption, £110 4s.; Royal, for women and children, £82 10s.; Poplar, for accidents, £80 9s.; West London, £78 16s.; London Homœopathic, £78 14s.; Royal Westminster Ophthalmic, £72 10s.; Royal Orthopædic, £72 6s.; Central London Throat and Ear, £69 14s.; Miller Memorial, £67 9s.; National for Diseases of the Heart and Paralysis, £61 18s.; Evelina for Sick Children, £58 11s.; London Temperance, £56 15s.; Hospital for Diseases of the Throat, £58 13s.; West End, for diseases of the nervous system, £57 18s.; Royal South London Ophthalmic, £56 15s.; St. Mark, for fistula, £55 8s.; Hospital for Women, £57 14s.; Hospital for Diseases of the Skin, £53 6s.; Central London Ophthalmic, £51 6s.; Lock Hospitals, £53 17s.; etc. The highest awards to dispensaries were: Infirmary for Consumption, £97 6s.; Western General, £42 3s.; West Ham, Stratford, &c., £41 9s.; Chelsea, Brompton, &c., £40 18s.; Surrey, £40 12s.; Stamford Hill, Stoke Newington, &c., £36 6s.; Westminster General, £33 12s.; Islington, £35 1s.; Farringdon General, £32 14s.; Finsbury, £33 1s.; Metropolitan, £33 1s.; Kensington, £31 15s; Queen Adelaide, £31 17s.; Royal South London, £30 7s.; South Lambeth, Stockwell, &c., £29 18s.; Public, £29 12s.; Holloway and North Islington, £27 7s.; &c. The largest awards to convalescent homes were: Metropolitan, £236 14s.; Morley House, £141 10s.; Mrs. Gladstone's Free, £95 15s.; Bexhill, £85; St. Andrew's, Clewer, £79 8s.; St. Leonard's, £70 7s.; Herbert, Bournemouth, £55 17s.; St. Andrew's, Folkestone, £50 8s.; King's College, Hemel-Hempstead, £48 9s.; London and Brighton, £45 15s.; Children's Convalescent Homes, Ramsgate and Redhill, £31 19s.; Princess Frederica's, £30 18s.; £500 was awarded the Hospital Surgical Appliance Committee; £91 1s. the Surgical Aid Society; and £72 12s. the Provident Surgical Appliance Society.

